# Multi-Point Wireless Temperature Sensing System for Monitoring Pharmaceutical Lyophilization

**DOI:** 10.3389/fchem.2018.00288

**Published:** 2018-07-17

**Authors:** Xiaofan Jiang, Tong Zhu, Tatsuhiro Kodama, Nithin Raghunathan, Alina Alexeenko, Dimitrios Peroulis

**Affiliations:** ^1^School of Electrical and Computer Engineering-Birck Nanotechnology Center, Purdue University, West Lafayette, IN, United States; ^2^School of Aeronautics and Astronautics, Purdue University, West Lafayette, IN, United States; ^3^Formulation Technology Research Laboratories, Daiichi Sankyo Co., Ltd., Hiratsuka-shi, Kanagawa, Japan

**Keywords:** freeze drying, lyophilization, PAT, wireless sensor networks, energy harvesting, sublimation tracking, process optimization, sublimation rate

## Abstract

This work presents the design and evaluation of a fully wireless, multi-point temperature sensor system as a Process Analytical Technology (PAT) for lyophilization. Each sensor contains seven sensing elements which measure the product temperature at various positions of the contents of a glass vial. The sensor performance was studied by freeze drying experiments with sensor placement in both center and edge of full shelf of 6R glass vials with 4 ml fill volume. Product temperature profile and primary drying time measured at the bottom center position in the glass vial by the wireless sensor as well as the primary drying time are in close comparison with the thermocouple data. The drying times during primary drying were determined at the top, higher middle, lower middle and bottom positions which are 3.26 mm apart vertically in the vial by the wireless sensor based on the temperature profile measured at different positions. For a center vial, the drying time from the start of primary drying to each layer was measured at 3.9, 9.3, 14.2, and 21 h respectively, allowing to track the sublimation interface during primary drying phase. In addition, sublimation rate at each layer was calculated based on the drying time and theoretical weight loss of ice in the product. The sublimation rate at the beginning of the primary drying was similar to the sublimation rate by gravimetric method. Furthermore, the vial heat transfer coefficient (*K*_*v*_) was also calculated based on the sublimation rate. Thus, allowing the use of the multi-point wireless sensor to rapidly monitor the sublimation rate and *K*_*v*_ for every batch as continuous process verification. Similar tests were also conducted with 3% w/v mannitol solutions and the results were consistent demonstrating potential for real-time monitoring, process verification and cycle optimization for pharmaceutical lyophilization.

## 1. Introduction

Lyophilization, or freeze-drying, is a commonly used and well-established process to preserve the original structure of a heat sensitive biological and/or pharmaceutical product (e.g., Anti-body, peptides, vaccines, etc.) during drying and, moreover, during long-term storage (extending the shelf life of pharmaceutical formulations). Freeze-drying involves ice removal from a frozen product at low pressure through a sublimation process. It was reported by the Food and Drug Administration (FDA) that about 50% of over 300 FDA and EMA approved biopharmaceutical products are freeze-dried (Corver et al., 2017).

A typical freeze-drying cycle consists of three steps. First, the solution is completely solidified during the freezing step. In the following step, denoted as primary drying, the pressure in the drying chamber is reduced and the shelf temperature is elevated to allow sustaining ice sublimation. After the initial ramping phase to the desired shelf set-point, the heat supplied by the shelves and the removal of heat by sublimation is balanced and the system is in steady state. In this initial drying phase, the majority of the water in the material is sublimated. The last step, denoted as secondary drying, aims to remove unfrozen water molecules, since the ice was removed in the primary drying phase. In this phase, the shelf temperature is raised higher than in the primary drying phase to break any physico-chemical interactions that have formed between the water molecules and the frozen material. Product temperature monitoring during a freeze-drying cycle is traditionally performed using a single-point sensor such as thin wire thermocouples and resistance thermal detectors (RTD, PT-1000) in a lab environment. Variations across freeze dryers as well as the spatial distribution of vials inside a given freeze dryer often result in substantial differences in the vials heat transfer coefficients and temperature profiles. While such differences may be tolerable in laboratory-scale experiments, they can cause considerable complications in production-level machines. Therefore, accurate process condition monitoring is becoming increasingly critical in the freeze drying industry. However, the product temperature monitoring of thermocouples is limited because the wired thermocouples operation for temperature mapping (particularity in center position) on shelves is complicated at a production scale freeze dryer. In 2008, an innovative wireless sensor was reported (Schneid and Gieseler, 2008) which has great potential PAT tool in a large scale freeze dryer.

Sublimation rate is an important indicator for optimizing shelf temperature and chamber pressure during primary drying. In addition, the vial heat transfer coefficient (Kv) is also critical particularly for understanding differences between laboratory and commercial production-level freeze dryers. It is also useful when studying heat transfer rates derived from glass vials (primary drying conditions, Pikal et al., 1984). Conventional techniques for evaluating the sublimation rate and the vial heat transfer coefficient in lyophilization runs employ the gravimetric method which measures the solution weight before and after freeze drying (Hottot et al., 2005). However, the gravimetric method is a complicated operation and needs extra-batches for measuring these quantities. Furthermore, to perform the gravimetric analysis, the primary drying process must be stopped before the end of the primary drying process. Such partial runs do not allow us to evaluate the endpoint of primary drying time, cake appearance, and water content in the same batch.

In our previous work we demonstrated multi-point wireless temperature sensors for monitoring product temperature using pure water (Raghunathan et al., 2015, 2016). In this work, we evaluate practically-important solutions based on sucrose and d-Mannitol. These are representative bulking agents constituting different characteristic cakes (Johnson et al., 2002). Moreover, we expand on the abilities of the wireless multi-point sensors to track the sublimation process and to simultaneously determine the sublimation rate and the vial heat transfer coefficient by utilizing the Multi point temperature sensing system. This paper also discusses the potential of the wireless multi-point sensors for real-time monitoring, process verification and cycle optimization for pharmaceutical lyophilization in laboratory process development as well as when scaling to pilot or production scale.

## 2. Materials and methods

### 2.1. Sensor operation principle

**Figure 1:**
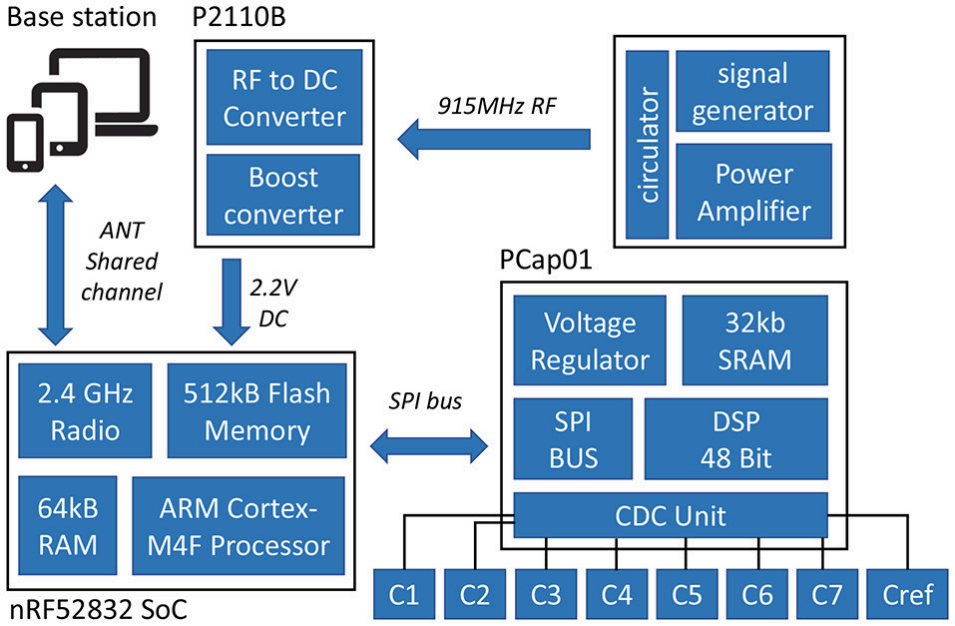
Block diagram of the multi-point wireless sensor system.

The wireless multi-point temperature sensor system used in this study was similar to the system designed by Raghunathan et al. (2015, 2016) with some functionality upgrades including both sensor hardware design changes as well as software improvements. The tested system consisted of 8 sensors, a PC base station, and an Radio frequency (RF) signal generator (Mini-Circuits®, USA). Figure 1 shows the block diagram of the multi-point wireless sensor system.

Each sensor was comprised of three printed circuit boards (PCBs): (1) Capacitive Temperature Sensing probe (2) Micro-controller and Wireless Transceiver Module (3) RF energy harvester module for wireless powering. The sensing probe and PCB with microcontroller was connected by a board-to-board connector and the RF energy harvester was connected via a ribbon cable. This modular design increases the sensor's compatibility with different sensor probes. The whole sensor assembly occupies a footprint of 10.30 × 28.75 mm and the footprint of the RF harvester module was 21.84 × 21.72 mm.

#### 2.1.1. Capacitive temperature sensing probe

In order to effectively monitor the product temperature during lyophilization process, a probe with seven temperature sensing elements was designed to capture the temperature profiles at different levels of the product (Figure 4A). The sensing elements were the GRM series ceramic chip capacitors with a base capacitance of 1,200 pF with U2J dielectric that exhibits a linear temperature coefficient characteristic of −750±120 ppm/K in free space ^1^. The capacitive sensors were read using the PCap01 capacitance measurement circuit (ACAM, 2014). PCap01 utilizes an RC discharge measurement circuit (TDC) to measure seven temperature sensing capacitors and one reference capacitor with attofarad (aF) resolution. The measured values were recorded as a ratio of the discharge times of the sense capacitors and a known reference capacitor. For this application, the reference capacitor was chosen to be a 1,200 pF, NP0-dielectric-based capacitor, which has a temperature coefficient of ± 30 ppm/K. The recorded values were read via a serial interface to the micro-controller.

**Figure 2:**
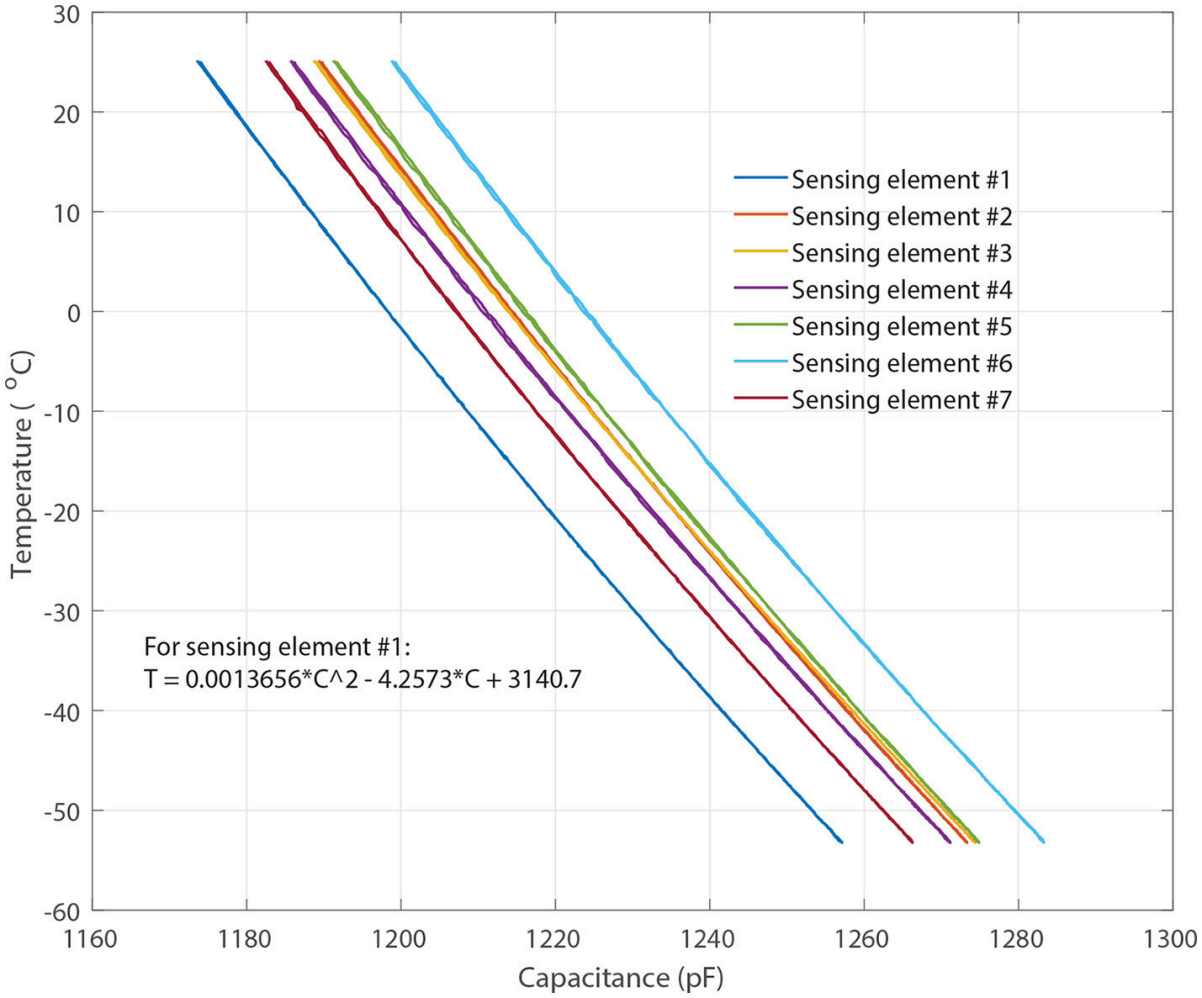
Measured capacitance vs. temperature for each of the sensing elements where Sensing elements #1–7 represent the capacitive sensing elements shown in Figure 4A.

Each of the sensing probes was encapsulated with 15 micrometer parylene-C coating to electrically insulate and protect it from cryogenic shocks (Figure 4). A layer of sliver Radio-frequency interference (RFI) shield coating (MG-Chemicals, 2018)^2^ was also applied to the sensor probe to protect the sensor from RF interference. The sensors were then individually calibrated using thin wire thermocouples (36-gauge, Omega, Newport, CT, USA) in an environmental chamber from 20° to −55^o^C. Figure 2 shows the measured capacitance vs. temperature for each of the sensing elements in air indicating each of the elements has a predictable monotonic response. The general temperature-capacitance relationship is given by:

(1) $$\begin{array}{l} T=A\times {C_{x}}^{2}-B\times C_{x}+D \end{array}$$

where *C*_*x*_ is the capacitance of each of the sensing elements in picoFarad (pF), and *T* represents temperature in Celsius (°*C*). The coefficients (A,B and D) of sensing elements #1 are shown in Figure 2 and the other elements can be extracted in a similar fashion. The calibration equations were stored in the micro-controller for direct transmission of the temperature to the base station.

#### 2.1.2. Micro-controller and wireless transceiver module

Similar to previous work, the nRF52832 system on chip^3^ was employed to process and transmit the measurements from PCap01. It features a very low power 32-bit ARM Cortex-M4F processor with a built-in-radio that operates in the 2.4 GHz ISM band which can output up to +4 dBm power and 512 kB flash storage for data-logging when the sensor is disconnected from the network. The ANT wireless communication protocol was used for communication between sensors and the base station. ANT is an ultra low power RF protocol implementing short range (20–30 feet in free space) and low data rate (up to 1 Mb/s). For this application, the bi-directional ANT shared channel topology was used with the capability to connect 60 leaf-nodes (sensors) with one tree- node (base station).

#### 2.1.3. RF energy harvester

An RF to DC to energy harvester was used to ensure a battery free operation. The energy harvester device used is the P2110B^4^ interfaced with a 915 MHz surface-mount technology (SMT) antenna. The device harvests 915 MHz RF energy and stores it in a external capacitor. In this system, an 85-mF super-capacitor with low discharge current and low equivalent series resistance (ESR) of 80 Ω was used. The capacitor's compact size (20 × 18 × 1 mm^3^) also made it an ideal selection for this application. The harvester outputs a user-defined voltage on the output pin once the capacitor charging threshold has been reached. The output voltage is maintained until the voltage falls below the internally set low-voltage threshold for the capacitor. The system operates down to −15 dBm of input power with an output current of 50 mA with 55% RF to DC conversion efficiency.

#### 2.1.4. Base station

**Figure 3:**
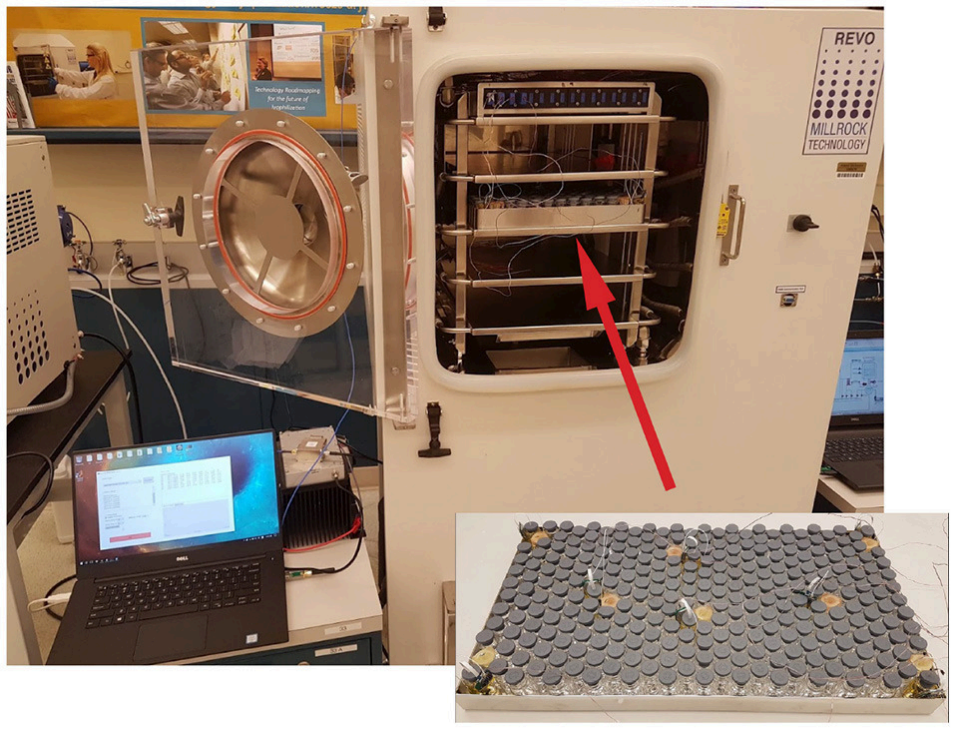
Typical arrangement of the vials with sensors and thermocouples in the tray and into the freeze dryer.

For this application, a custom PC application (Figure 3) was developed in C# with .NET framework which works with an external ANT radio USB stick. The application automatically adds available devices to the channels and outputs the received data to local storage. It also features the real-time graphing of the received data. Since ANT is not natively supported by PC, a custom USB stick with an 8-channel ANT Connectivity integrated circuit (IC) (nRF24AP2-USB) was used to enable the ANT communication.

### 2.2. Freeze drying setup

Freeze-drying was performed in a laboratory-scale freeze-dryer (REVO, Millrock Technology, Kingston, NY) equipped with a vacuum capacitance manometer and pirani gauge pressure sensor located at Purdue university. A 915 MHz monopole antenna was mounted on the side of the chamber for wirelessly powering the sensor. In addition, to prevent leaks and protect the coaxial cable from vacuum during freeze drying, a custom vacuum feed-through SMA connector was used to pass the RF coaxial cable inside the chamber to power the antenna. The computer with 2.4 GHz ANT connectivity USB stick for sensor operation and data collection was placed next to the freeze-dryer.

Five freeze drying runs were performed to evaluate the sensors' measurement accuracy, sublimation tracking ability, determination of sublimation rate and the heat transfer coefficient (*K*_*v*_) (Table 1). Pre-defined freeze drying recipes were used in all of the five runs based on each product's properties in 6R SCHOTT® pharmaceutical vials with 4 ml fill volume loaded on one entire shelf (360 vials). D-mannitol and sucrose were purchased from Sigma (Sigma Chemical Company, Germany) and used as received with ultra pure water. One of the runs (cycle 1) in partial completion was performed with ultra pure water for measuring the vial heat transfer coefficient (*K*_*v*_) of the REVO lyophilizer. Two of the runs (cycle 2 and 3) were performed using 5% w/v sucrose solution and two additional runs (cycle 4 and 5) were performed using 3% w/v mannitol solution. In each experiments, eight wireless sensors were placed across the entire shelf for a complete shelf mapping. In addition, eight 36-gauge wire thermocouples were placed in the vials adjacent to vials containing wireless multi-points sensor for comparison. Each wireless multi-point sensor was placed in the center position touching the bottom of the vial. Similarly, the thin wire thermocouples were in the “center bottom" position to ensure adequate comparison of the temperature profiles between sensing elements of the wireless sensor and the thermocouple. Furthermore, during the freezing step of all four experiments, controlled nucleation (performed with Millrock FreezeBooster® at −5°C) was applied to reduce batch to batch as well as vial to vial variabilities.

**Table 1:** List of all freeze drying experiments.

**Cycle ID**	**Description**	**Goal**	**Recipe**	***K*_*v*_ (cal/s/K/cm^2^)**
1	pure water run	to estimate *k*_*v*_	Table 5	(3.4 ± 0.68) E^−4^
2	5% w/v sucrose (100% dried run)	the main run for studying the wireless sensor's ID capability	Table 6	(3.3) E^−4^
3	5% w/v sucrose (50% dried run)	to estimate the sublimation rate gravimetrically	Table 6^*^	*N*/*A*
4	3% w/v mannitol (100% dried run)	complementary study for the sublimation tracking	Table 7	(3.7) E^−4^
5	3% w/v mannitol (50% dried run)	to estimate the sublimation rate gravimetrically	Table 7^**^	*N*/*A*

* with 9 h primary drying time

** *with 5 h primary drying time*.

### 2.3. Sublimation tracking and end-point detection

Previous studies suggest that for most vials, the primary drying during the freezing process is a top-down process with a well-defined sublimation front moving through the product as it dries and to a lower degree, from the side to the center of the vial (Schelenz et al., 1995). Above the ice surface interface is dried product (commonly known as cake), below the interface is frozen product with ice crystals still remaining to be sublimed (Barley, 2009). As the primary drying process progresses, the different sensing elements on the sensing probe will start to lose contact with ice as the sublimation front moves from top to bottom, resulting in a sharp increases in slope in the temperature profile (Mascarenhas et al., 1997; Wouwer et al., 2014). End-point of the primary drying can be detected when the temperature at every sensing points converges together and the rate of temperature change was below 0.5°C/h.

### 2.4. Rapid determination of sublimation rate

**Figure 4:**
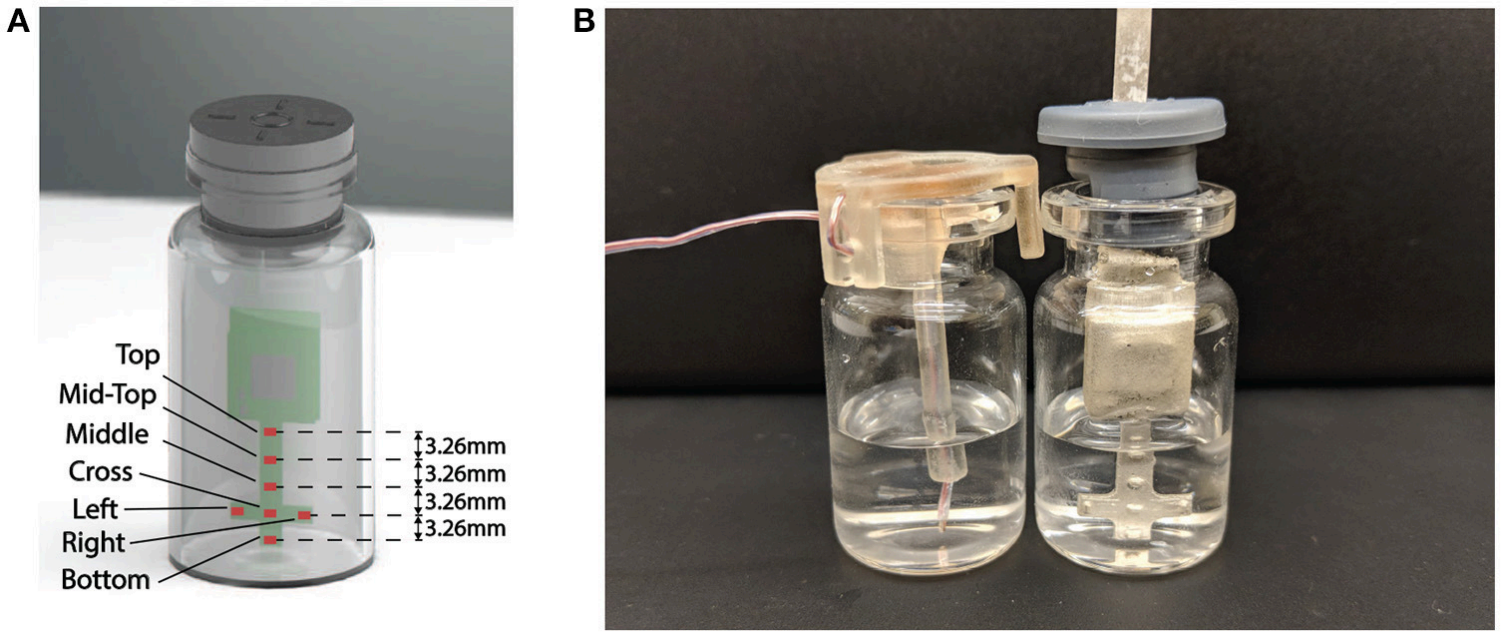
**(A)** Representation of the wireless multi-point sensor. Seven sensing elements are labeled. **(B)** Placement of Wireless multi-point sensor and thermocouple in the vial.

The sublimation rate is the most important factor for understanding the drying process and the quality of the freeze drying product. Since the major driving force for ice sublimation is the difference in ice vapor pressure between the product and the condenser, the ice sublimation rate can be influenced by product temperature and chamber pressure (Koganti et al., 2011). Traditionally the sublimation rate was measured gravimetrically (Pikal et al., 1984; Pisano et al., 2011) by repeatedly weighting vials before and after the freeze drying process or using tunable diode laser absorption spectroscopy (TDLAS) in the laboratory scale (Gieseler et al., 2007). This is an added cost to the production run since additional batch for gravimetric testing is required. However, since the wireless multi-point sensor is capable of tracking the sublimation front and measuring drying time up to each sensing element location in the product, the entire primary process can be decomposed into the consecutive drying of each “layer of frozen product (of 3.26 mm in thickness, Figure 4A)” between different sensing elements. Then the sublimation rate could be calculated based on the drying time (Table 2) and theoretical weight loss of ice in each layer of the product. The theoretical weight loss can be calculated based on the inner area of the vial and distance between the sensing elements as described in Equation 2:

(2) $$\begin{array}{l} \frac{dm}{dt}=\frac{A_{p}\;\cdot \;\Delta h_{sensor}\;\cdot \;\rho_{ice}\;\cdot \;\varepsilon }{t_{drying}} \end{array}$$

where *A*_*p*_ is the cross sectional area of the product, Δ*h*_*sensor*_ is the distance between sensing elements and *t*_*drying*_ is the drying time between sensing elements and ε = (1 − *C*_*solid%*_) is the porosity of the cake. Table 3 shows the calculated sublimation rate for each drying layer between the sensing elements of the product during the sucrose experiment.

**Table 2:** Primary drying time measured by multi-point wireless sensor.

	**Center vial (cycle 2)**		**Edge vial (cycle 2)**		**Center vial (cycle 4)**	
**Location**	**Drying time (h)**	**Time between layers (h)**	**Drying time (h)**	**Time between layers (h)**	**Drying time (h)**	**Time between layers (h)**
Middle-Top	3.9	*N*/*A*	3.5	*N*/*A*	3.3	*N*/*A*
Middle	9.3	5.4	7.7	4.2	6.1	2.8
Cross	14.2	4.9	12.5	4.8	8.4	2.3
Bottom	21	6.8	17	4.5	11.4	3.0

**Table 3:** Comparison between sublimation rate measured by multi-point wireless sensor and gravimetric method.

	**Center vial (Cycle 2 and 3)**		**Edge vial (Cycle 2 and 3)**		**Center vial (Cycle 4 and 5)**	
**Location**	**Sensor**	**Gravimetric**	**Sensor**	**Gravimetric**	**Sensor**	**Gravimetric**
Top-Mid to Middle	0.17 (g/h)	0.16 (g/h)	0.21 (g/h)	0.22 (g/h)	0.33 (g/h)	0.39 (g/h)
Middle to Cross	0.18 (g/h)	*N*/*A*	0.19 (g/h)	*N*/*A*	0.40 (g/h)	*N*/*A*
Cross to Bottom	0.14 (g/h)	*N*/*A*	0.20 (g/h)	*N*/*A*	0.30 (g/h)	*N*/*A*

### 2.5. Rapid determination of vial heat transfer coefficient (*K*_*v*_)

Vial heat transfer coefficient (*K*_*v*_) describes the relationship between heat flow and temperature difference during primary drying and needs experimental measurements (Pikal et al., 1984). There are three major contributions to *K*_*v*_: direct conduction from the shelf to the vial bottom, conduction through the vapor between the vial bottom and the shelf, and radiative heat transfer. Consequently, heat transfer coefficient will vary from vial to vial for a same batch and it is a crucial parameter to monitor for successful freeze drying. Traditionally, a separate trial run is required to evaluate the *K*_*v*_ for commercial production. However, with the ability to measure the sublimation rate during primary drying, the multi-point wireless sensor is also capable of estimating the heat transfer coefficient (*K*_*v*_) using the following equation:

(3) $$\begin{array}{l} K_{v}=\frac{dm}{dt}\;\cdot \;\frac{\Delta H_{sub}}{<T_{shelf}-T_{b}>\;\cdot \;A_{v}} \end{array}$$

where $\frac{dm}{dt}$ is the calculated sublimation rate between two sensing points, “<…>" denotes an average over the primary drying phase, Δ*H*_*sub*_ is the latent heat of sublimation for water and *A*_*v*_ is the the cross-sectional vial area.

## 3. Results and discussion

### 3.1. Compatibility study of the multi-point wireless sensor and thermocouple

**Figure 5:**
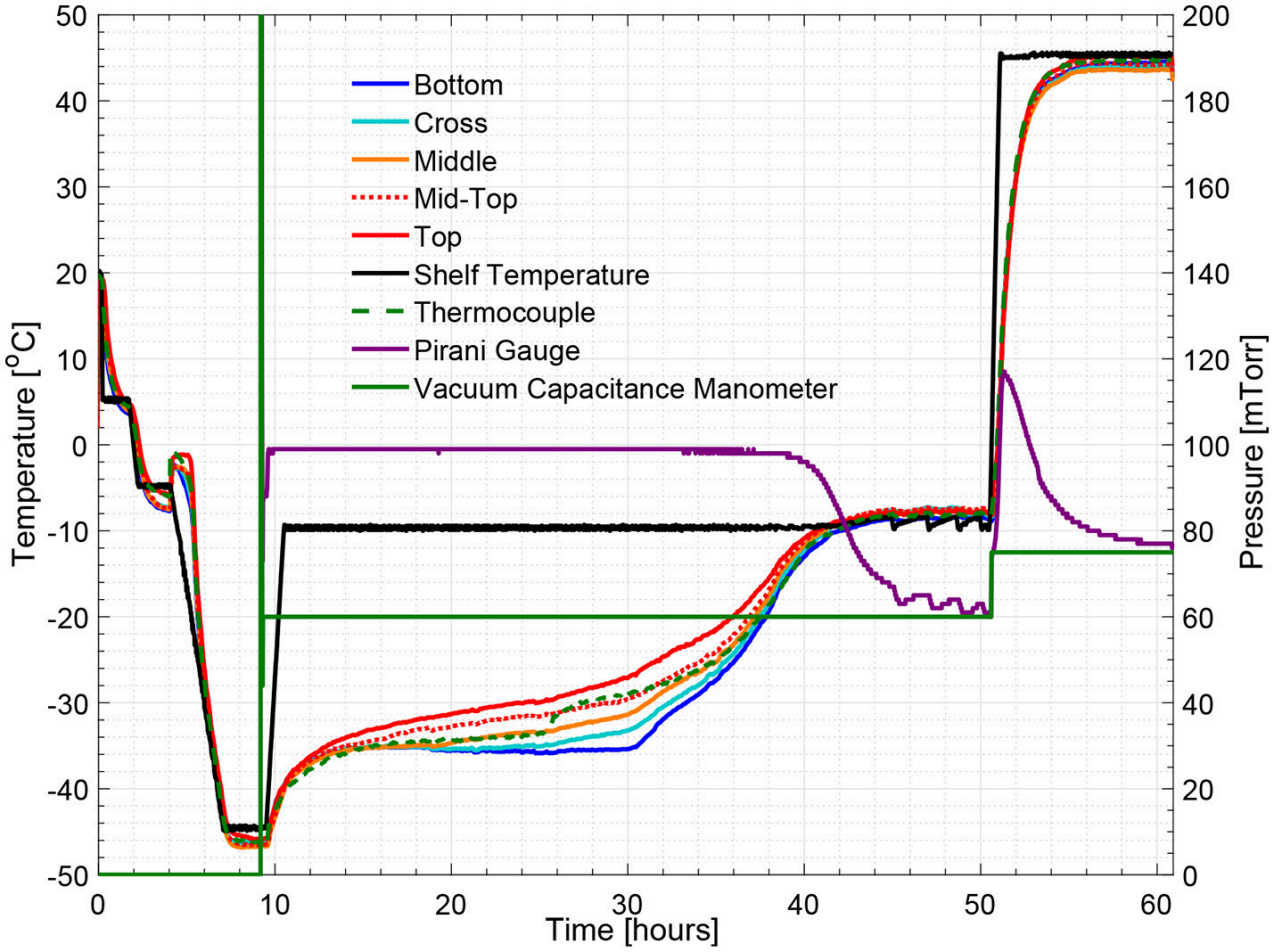
Measured temperature and pressure over time profile of 5% w/v sucrose solution for a center vial (cycle 2).

**Figure 6:**
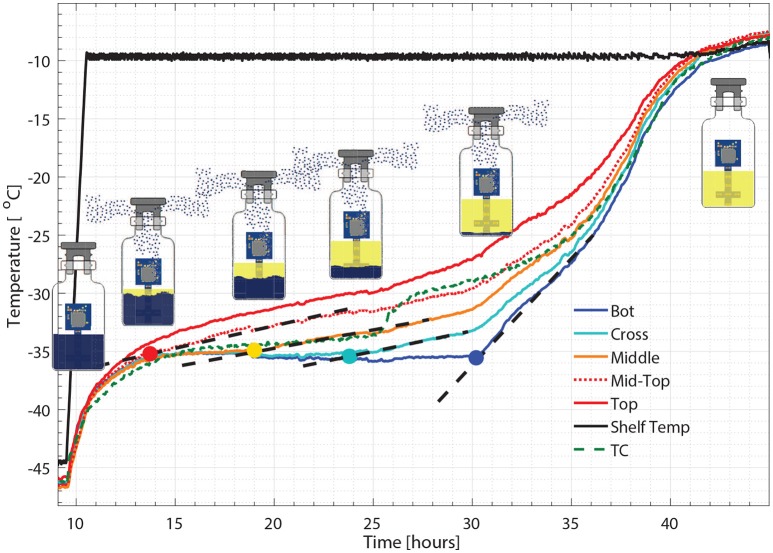
Zoomed-in figure for primary drying phase Figure 5, where each colored circle indicates the crossing point for the sublimation front with corresponding sensing point and the dash line represents the change of slope.

**Figure 7:**
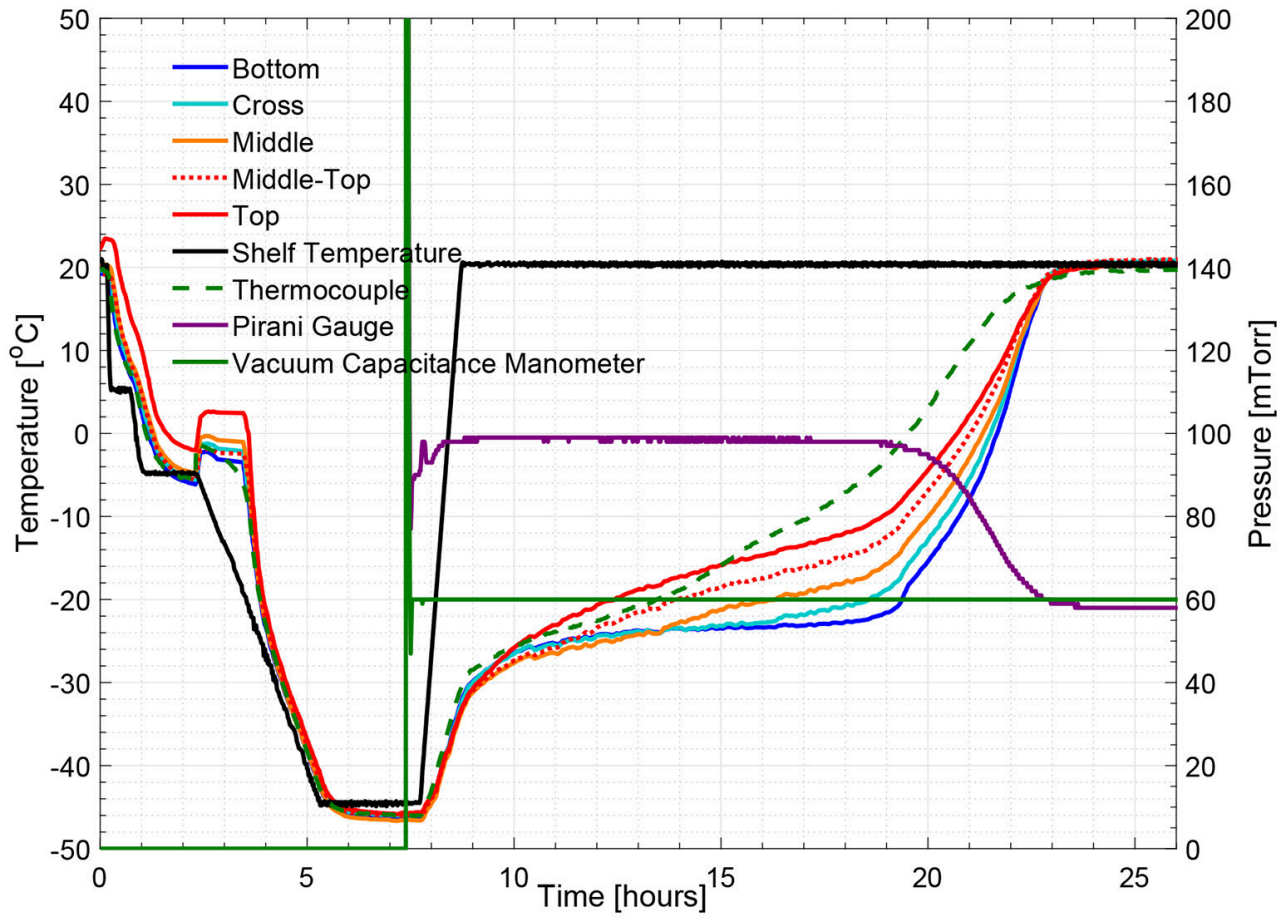
Measured temperature and pressure over time profile of 3% w/v mannitol solution (cycle 4).

Figure 5 illustrates the temperature histories during an entire freeze drying process for a 5% w/v sucrose solution, monitored by one wireless multi-point sensor placed in a representative center vial and one 36-gauge thermocouple placed in an adjacent vial. Figure 6 illustrates the primary drying phase for the 5% w/v sucrose solution run (Cycle 2) shown in Figure 5 and indicates the crossing point of the sublimation front for each sensing element. The shelf temperature was measured by using a built-in thermocouple that was previously installed into the freeze dryer. Five out of seven temperature profiles measured by wireless multi-point sensor are color coded from red to blue lines shown in Figure 5 representing sensing elements from top to bottom. All seven sensing elements showed good agreements with the adjacent thermocouple during the freezing step with a maximum temperature difference observed before nucleation (right before 4 h) of 1°C. After the freezing step, at around 10 h, primary drying started with the ramp up of the shelf temperature to −10°C. At the end of the primary drying step, the temperature profiles measured by the wireless multi-point sensor and the thermocouple are in good agreement and reach a steady state temperature at the same time at around 45 h. In addition, it is worth mentioning that the temperature reading from the bottom sensing elements of the multi-point wireless sensor showed the best agreement with the thermocouple placed in the bottom center in the adjacent vial during steady state for the entire process since they were both placed in the bottom center of the neighboring vials (assuming identical product resistance, *R*_*p*_, due to controlled nucleation). Similarly, a more aggressive freeze drying experiment with 3% w/v mannitol solution was conducted as well, and the results supported the same conclusions (Figure 7). This result suggests that the multi-point wireless sensor is capable of measuring reliable and accurate temperature.

### 3.2. Sublimation tracking and end-point detection

**Figure 8:**
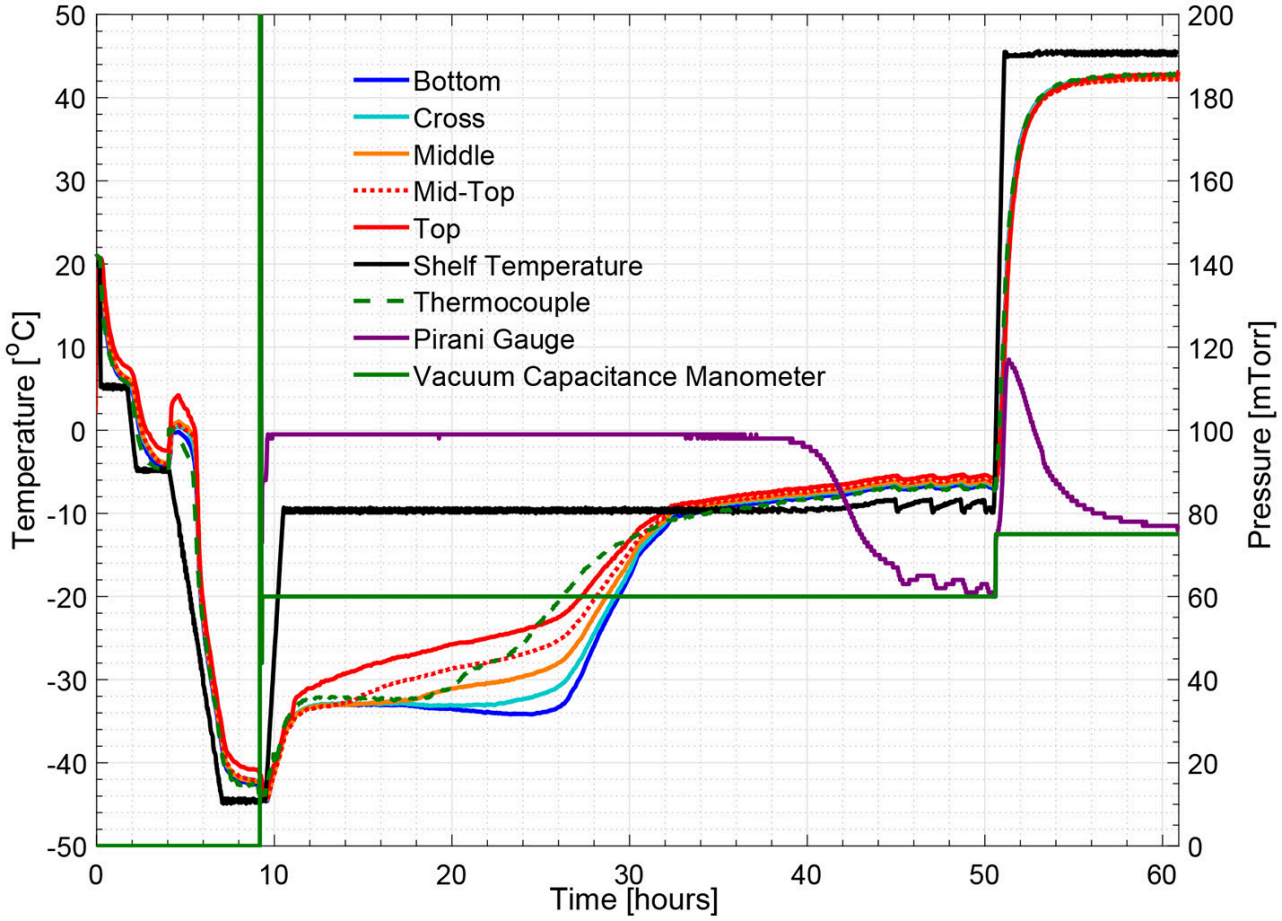
Measured temperature and pressure over time profile of 5% w/v sucrose solution for an edge vial (cycle 2).

Figures 5, 8 show respectively the complete temperature profile of the experiment with 5% w/v sucrose solution (cycle 2) for center and edge vials containing multi-point wireless sensors. It can be seen that drying proceeded from top to bottom as each sensing element's temperature profile “departed” from the “bottom temperature” in the same order.

As seen from Figure 6, a zoom-in figure showing the primary drying step of the center vial during the experiment mentioned above, the sublimation front was clearly tracked as each sensing elements lost contact with ice crystals. Therefore, the drying time at the mid-top, middle, cross and bottom positions in the vial during primary drying could be determined by the wireless sensor based on the temperature profile measured at different positions. Specifically, a change in temperature rising rate is identified with the slope of tangent dashed line for each sensor location as shown in the figure. The “anchor point (colored circles)" of a tangent line on the bottom location temperature profile is identified as the end of partial drying for each layer. For the center vial shown in Figure 6, the drying time from the start of primary drying to each layer were shown in Table 2. Similarly, the measured drying time for the sensor placed in the edge vial during the same run were also shown in Table 2. In addition, the end-point of the primary drying were detected at 43 and 34 h for center and edge vials respectively, also in excellent agreement with the adjacent thermocouples. Furthermore, the end-point of primary drying for the center vial agrees well with the onset of convergence of the capacitance manometer and Pirani gauge pressure sensors indicating that the center vial was the last to finish the primary drying. In addition, a heat and mass transfer simulation of the primary drying in a vial with the same experimental parameters was conducted (Pikal, 1985). Specifically, *K*_*v*_ was obtained from the gravimetric study of cycle 1. The product resistance, *R*_*p*_ as a function of the cake length is computed from temperature history of the bottom sensing element in cycle 2. This is then fitting to the standard empirical form Pikal et al. (1983) of:

(4) $$\begin{array}{l} Rp\left(L_{ck}\right)=R_{0}+\frac{A_{1}\;\cdot \;L_{ck}}{1+A_{2}\;\cdot \;L_{ck}}\left({\text{cm}}^{2}\text{-Torr-hr/g}\right) \end{array}$$

where *R*_0_ = 0, *A*_1_ = 71.55 (cm-Torr-h/g), *A*_2_ = 34.1 (cm^−1^) are fitting coefficients found for the best curve fit. *L*_*ck*_ (cm) is the cake length. Finally, these parameters are used to model the temperature at all the locations of the sensing elements for cycle 2. Table 4 shows the comparison between experimental and simulated results for the drying time between each layer of the center vial. The results showed excellent agreement between the experimental and simulated results.

**Table 4:** Comparison between experimental and simulation primary drying time for cycle 2.

**Sensor location**	**Experimental results (h)**	**Simulation results (h)**	**Difference (%)**
Middle-Top	0	0	*N*/*A*
Middle	5.4	5.1	4.8
Cross	10.3	10.3	-0.1
Bottom	17.1	15.2	11.1

**Table 5:** Freeze drying recipe used for 5% w/v sucrose solution.

	**1**	**2**	**3**	**4**	**5**	**6**	**7**	**8**
**Freezing step**								
Shelf setpoint [°C]	20	20	5	5	–5	–5	–45	–45
Time [min]	0	10	0	90	30	90	180	120
**Primary/Secondary drying**								
Shelf setpoint [°C]	–45	–10	–10	45	10			
Time [min]	15	60	2,400	720				
Vaccum setpoint [mTorr]	60	60	60	75				

**Table 6:** Freeze drying recipe used for 3% w/v mannitol solution.

	**1**	**2**	**3**	**4**	**5**	**6**	**7**	**8**
**Freezing step**								
Shelf setpoint [°C]	20	20	5	5	−5	−5	−45	−45
Time [min]	0	10	0	90	30	90	180	120
**Primary drying**								
Shelf setpoint [°C]	−45	20	20					
Time [min]	5	60	1800					
Vaccum setpoint [mTorr]	60	60	60					

**Table 7:** Freeze drying recipe used for pure water.

	**1**	**2**	**3**	**4**	**5**	**6**	**7**	**8**
**Freezing step**								
Shelf setpoint [°C]	20	20	5	5	−5	−5	−45	−45
Time [min]	0	10	0	90	30	90	180	120
**Primary drying**								
Shelf setpoint [°C]	−45	−10	−50	−50				
Time [min]	15	180	0	9999				
Vaccum setpoint [mTorr]	60	60	60	60				

### 3.3. Rapid determination of sublimation rate

As previously discussed in Section Rapid Determination Of Sublimation Rate, the sublimation rate can be determined based on the drying time between each layer. The multi-point wireless sensor is capable of measuring in real-time the sublimation rate at various positions during the freeze-drying cycle. For example, the sublimation rates for the center vial between mid-top to middle sensing point in cycle 2 can be calculated as follows:

(5) $$\frac{dm}{dt}=\frac{3.14\;{\text{cm}}^{2}\;\cdot \;0.326\text{ cm ​ }\cdot \;0.918\;{\text{g/cm}}^{3}\;\cdot \;95\%}{5.4\text{ h}}=0.17\text{ g/h}$$

Similarly, the sublimation rates at the same location in cycle 4 (3%w/v mannitol) can be calculated as follow:

(6) $$\frac{dm}{dt}=\frac{3.14\;{\text{cm}}^{2}\;\cdot \;0.326\text{ cm }\cdot \;0.918\;{\text{g/cm}}^{3}\;\cdot \;97\%}{2.8\text{ h}}=0.33\text{ g/h}$$

Table 3 shows the complete measured sublimation rates for cycle 2 and 4 using the multi-point wireless sensor. In order to verify this approach of rapid determination of the sublimation rate, two additional experiments with identical process and product setting (only the primary drying time is 50% shorter for valid gravimetric study) were performed (cycle 3 and 5) using the gravimetric method. Each vial with the muli-point wireless sensor was weighted before and after the drying. Table 3 shows the comparison between sublimation rates ($\frac{dm}{dt}$) calculated using the muli-point wireless sensor and gravimetric method. Comparing the initial stages of the primary drying with the prior completed drying run shows close agreement in the average sublimation rate for both typical formulation excipients (crystalline mannitol and amorphous sucrose), which suggests that the multi-point wireless sensor has great potential for rapidly determinating the sublimation rate. It is noted that the sublimation rate from the sensor is an average quantity over the drying time in each layer. However, it is sufficiently accurate since heat and mass transfer modeling results suggest that the sublimation rate changes relatively slowly during primary drying (with the only exception in the initial fast ramping stage). This additional real-time process monitoring technique may become instrumental in optimizing the freeze drying process on both laboratory- and production-scale machines.

### 3.4. Rapid determination of vial heat transfer coefficient (*K*_*v*_)

With the demonstrated capability to determine the sublimation rate using the sensor in the previous section, we proceed further by combining it with the temperature data to estimate the vial heat transfer coefficient without the need of any gravimetric study. The following wireless sensor measurements over the course of drying from the “Mid-top” to “Middle” sensing element locations from (cycle 2) can be used to estimate the heat transfer coefficient at the center position as follows:

(7) $$\frac{dm}{dt}=\;0.17\text{ g/h}$$

(8) $$<\;T_{sh}\;-\;T_{b}\;>=\text{​ }((-10{}^{\circ}\text{C})\;-\;(-35.1{}^{\circ}\text{C}))\;=\;25.1\;{}^{\circ}\text{C}$$

(9) $$K_{v}\;=\frac{0.165\text{ g/h}}{3,600\text{ s}}\;\cdot \;\frac{676\text{ cal/g}}{25.1\text{ K }\cdot \;3.8\;{\text{cm}}^{2}}=\;3.3\;\cdot \;10^{-4}{\text{cal/s/K/cm}}^{2}$$

Similarly, for the heat transfer coefficient at the center position from the full shelf 3% w/v mannitol solution run (cycle 4):

(10) $$\frac{dm}{dt}=\;0.33\text{ g/h}$$

(11) $$<\;T_{sh}\;-\;T_{b}\;>=\;((20{}^{\circ}\text{C})\;-\;(-24{}^{\circ}\text{C}))\;=\;44{}^{\circ}\text{C}$$

(12) $$K_{v}\;=\frac{0.33\text{ g/h}}{3,600\text{ s}}\;\cdot \;\frac{676\text{ cal/g}}{44\text{ K }\cdot \;3.8\;{\text{cm}}^{2}}=\;3.7\;\cdot \;10^{-4}{\text{cal/s/K/cm}}^{2}$$

Compared to the actual *K*_*v*_ = (3.4 ± 0.68) E^−4^ cal/s/K/cm^2^ previously measured from the center of the freeze drying experiment with ultra pure water. These estimations fall in the range of the actual *K*_*v*_ considering the cycle to cycle variation. A higher *K*_*v*_ measured in cycle 4 might have been caused by the higher shelf temperature during primary drying with a non-linear increase of the radiative heat transfer component. Table 1 shows the comparison between the estimated *K*_*v*_ by the wireless sensor (cycle 2 and 4) and the actual *K*_*v*_ at the center position of the batch. Combined with the results from the determination of the sublimation rate, the wireless multi-point sensor has great potential for continuous process verification, with the ability to optimize the process parameters for every batch. This is crucial especially in production scale, since additional batchs for gravimetric analysis are often required, increasing production cost. In some GMP facilities, gravimetric study of the heat transfer coefficient is unfeasible mostly due to the operational cost and the current sensor opens up an opportunity to have an estimation of *k*_*v*_ along with a normal product run.

## 4. Conclusions

The measured profile of product temperatures using the new multi-point wireless sensor during freeze-drying of manntiol and sucrose solutions showed excellent agreement with thermocouple as well as the simulated data. The sublimation progress during primary drying was clearly tracked by the multi-point wireless sensor. The sublimation rate extracted from the wireless multi-point sensors' measurement also showed good match with the actual grametric measurements. Furthermore, the vial heat transfer coefficient (*K*_*v*_) calculated from the measured sublimation rate by the multi-point wireless sensor was in excellent agreement with the actual gravimetrically measured vial heat transfer coefficient. In addition, the ability of multi-point sensing endows the wireless sensor with a more elaborate characterization of the freeze drying profile as demonstrated through the *end*−*point* *detection* and the *sublimation* *front* *tracking*. These results suggested the wireless multi-point temperature sensor system has a potential for real-time monitoring, process verification and cycle optimization for pharmaceutical lyophilization in laboratory process development and GMP production.

## Author contributions

NR and XJ designed the multi-point wireless sensor system. XJ and TK created and performed the freeze-drying experiments. TZ performed the heat and mass transfer simulation. DP and AA provided guidance and oversaw the entire study.

### Conflict of interest statement

The authors declare that the research was conducted in the absence of any commercial or financial relationships that could be construed as a potential conflict of interest.

## Footnotes

^1^(Murata, 2018) GRM Series General Purpose Monolithic Ceramic Capacitors

^2^SUPER SHIELDTM SILVER CONDUCTIVE COATING

^3^(Nordicsemi, 2018) nRF52832 ANT multiprotocol SoC

^4^(Powercast R®, 2018) 915 MHz RF Powerharvester® receiver
